# The relationship between trait anxiety and sleep quality in college students: an exploratory analysis of physical activity as a moderator

**DOI:** 10.3389/fpsyt.2025.1563237

**Published:** 2025-06-06

**Authors:** Liyang Zhong, Xiaochen Ma, Sen Li, Ling Yu

**Affiliations:** ^1^ Linhai College, Zhejiang Open University, Linhai, China; ^2^ School of Physical Education, Shanghai University of Sport, Shanghai, China; ^3^ School of Sports and Health, Shanghai Lixin University of Accounting and Finance, Shanghai, China

**Keywords:** physical activity, college students, trait anxiety, sleep quality, moderating effects

## Abstract

**Objective:**

This study aimed to examine the association between trait anxiety and sleep quality among college students and to assess whether different levels and components of physical activity (PA) moderate this relationship.

**Methods:**

A cross-sectional survey was conducted among 2,902 college students. Standardized instruments were used to evaluate trait anxiety, sleep quality, and PA levels. Moderation regression models were constructed to test interaction effects.

**Results:**

Trait anxiety was significantly associated with decreased overall sleep quality and impairments across all sleep subdimensions. PA level significantly moderated the relationships between trait anxiety and four dimensions of sleep: sleep quality, sleep latency, sleep efficiency, and daytime dysfunction. Stronger buffering effects were observed under conditions of high intensity, long duration, and high frequency of PA.

**Conclusion:**

High-intensity, long-duration, and high-frequency physical activity may help alleviate anxiety-related sleep disturbances in college students, exhibiting a clear dose–response effect. The findings support exercise as a non-pharmacological strategy for improving mental health.

## 1 Introduction

There is a close association among trait anxiety, sleep quality, and physical activity (1–3). Studies have shown a bidirectional relationship between sleep quality and anxiety (4, 5)—anxiety can trigger various sleep problems, while poor sleep quality is a significant risk factor for anxiety onset (6), further aggravating internalizing symptoms and creating a vicious cycle. As one of the most common psychological disorders, anxiety is prevalent among college students, with detection rates significantly higher than those in the general population (7). In China, the anxiety detection rate among university students continues to rise, reaching 31% (8). Trait anxiety refers to a relatively stable tendency to experience anxiety over time (9). Abnormal emotional processing and neural activity patterns—such as dysfunctions in the anterior insula and dorsal anterior cingulate cortex—may heighten sensitivity to threat-related stimuli (10, 11). Elevated anxiety levels promote the release of stress-related hormones such as adrenaline and cortisol, disrupt central nervous system functioning, and lead to neurotransmitter imbalances (e.g., glutamate, GABA), all of which impair sleep regulation (12, 13). These physiological disruptions have been shown to correlate closely with impaired sleep quality (e.g., decreased sleep efficiency and prolonged sleep latency) (14). According to the biopsychosocial model (15), individuals with high trait anxiety often experience chronic physiological hyperarousal, which interferes with sleep through both psychological pathways (e.g., persistent worry) and biological mechanisms (e.g., dysregulated cortisol rhythms). The combination of anxiety and poor sleep quality is also associated with worse treatment adherence, a higher risk of acute deterioration, and increased mortality rates (16). Moreover, recent neuroimaging studies have revealed that sleep deprivation can enhance neural reactivity in emotion-related brain regions (e.g., the amygdala and insula) in individuals with high trait anxiety, thereby exacerbating the anxiety–sleep disturbance feedback loop (17).

Meanwhile, physical activity has been widely recognized as an effective strategy for emotional regulation (18). Prior research suggests that trait anxiety may impair sleep quality via heightened stress responses and disrupted neuroregulatory mechanisms, such as HPA axis dysregulation and neurotransmitter imbalance (e.g., glutamate and GABA) (19, 20). Conversely, physical activity helps mitigate anxiety and improve sleep through both stress-buffering and neurobiological pathways (21, 22). Observational studies and meta-analyses (23, 24) have shown that regular moderate-to-vigorous exercise is associated with reduced anxiety onset and enhanced sleep quality (25–27). Exercise contributes to emotional and sleep enhancement through multiple mechanisms, such as regulating the hypothalamic–pituitary–adrenal (HPA) axis, increasing serotonin and endorphin levels, and reducing stress hormones like cortisol (28–30). Ample evidence also suggests that regular physical activity improves sleep efficiency, shortens sleep latency, and increases total sleep duration (22, 31).

Although current research generally supports the positive effects of physical activity in improving anxiety and sleep, there is still no consensus on the optimal intervention format regarding exercise dosage—namely, intensity, frequency, and duration. Some studies suggest that moderate-intensity exercise is most effective (32), while others emphasize the advantages of high-intensity interval training (33, 34). Discrepancies in the effects of exercise duration, frequency, and type remain unresolved (35, 36). Moreover, sleep quality is a multidimensional construct, comprising sleep efficiency, latency, duration, and disturbances. Many existing studies have focused only on total PSQI scores, neglecting the possible heterogeneity between anxiety and different sleep subcomponents (37). In addition, little attention has been paid to the moderating role of demographic variables in these relationships. These limitations reduce the explanatory power of current evidence and hinder the development and dissemination of personalized intervention strategies across populations.

To address the above limitations, it is essential to further explore the specific pathways through which physical activity influences the relationship between trait anxiety and sleep quality. This study aims to fill these gaps from two perspectives. First, it will examine the associations between trait anxiety and various subcomponents of sleep quality to identify which aspects of sleep are most impaired among individuals with different levels of anxiety. Second, it will assess whether physical activity dosage moderates the relationship between trait anxiety and sleep quality, with particular attention to the moderating effects of exercise intensity, frequency, and duration. Given the heterogeneity of the college student population, key demographic variables will be controlled to enhance the explanatory power and generalizability of the findings. Unlike previous studies that have focused solely on overall sleep quality or simple correlations, this research employs a moderation model to systematically analyze the interaction mechanisms among the three variables.

Therefore, this study focuses on clarify the moderating role of physical activity in the relationship between trait anxiety and sleep quality among college students and to investigate how different levels of exercise dosage influence this effect. Specifically, the study proposes the following research questions and hypotheses: (1) college students with different levels of trait anxiety show significant differences in overall sleep quality and its subdimensions; (2) scores of sleep quality subcomponents are positively correlated with levels of trait anxiety; and (3) the intensity, frequency, and duration of physical activity significantly moderate the relationship between trait anxiety and sleep quality, with the strength of moderation varying by dosage. These hypotheses are grounded in neurobiological mechanisms described above, particularly the role of PA in modulating anxiety-related stress responses and sleep regulation. Based on a large-sample cross-sectional dataset and drawing upon previous research, this study aims to identify which subdimensions of sleep quality are impaired in students with high trait anxiety. It further seeks to elucidate how different levels, intensities, durations, and frequencies of physical activity modulate the association between trait anxiety and specific sleep components. Ultimately, the goal is to determine the most appropriate exercise dosage for students with high trait anxiety, ensuring the precision of exercise-based interventions, and to provide direction for clinical screening and targeted interventions, as well as a theoretical foundation for researchers and university administrators.

## 2 Research subjects and methods

### 2.1 Research subjects

This study employed a convenience sampling method to recruit participants from seven universities located in Songjiang University Town, Shanghai. Recruitment announcements were made via online campus platforms and class notifications, and all participants volunteered to take part in the study.

Inclusion criteria were as follows: individuals aged between 17 and 25 years, right-handed, non-sports major students, with no history of mental illness, and no recent use of barbiturates, benzodiazepines, or chloral hydrate. Additionally, participants were required to abstain from strenuous physical activity, as well as caffeine- or alcohol-containing beverages, within 24 hours prior to testing. To eliminate confounding factors potentially affecting sleep or anxiety outcomes, individuals were excluded if they had a history of head trauma, respiratory or cardiovascular diseases, or any recent sports injuries. All participants provided written informed consent after being informed of the procedures and potential risks.

### 2.2 Test procedure

Data were collected through paper-based questionnaires administered in designated classrooms or psychology laboratories at participating universities. All locations were well-lit, quiet, and enclosed. To minimize time-of-day effects on subjective evaluations, survey sessions were uniformly scheduled between 9:00–11:30 a.m. or 2:00–5:00 p.m. Each participant completed the questionnaire in a relatively private setting, with an average completion time of 15–20 minutes. Prior to the survey, investigators read standardized instructions aloud, explained each item, clarified that data would be used solely for scientific research, emphasized the importance of honest, independent, and voluntary responses, and informed participants of their right to withdraw at any time. To control for pre-sleep environmental influences, participants were instructed to “avoid electronic screen exposure (e.g., mobile phones, tablets, computers) for one hour before bedtime the night prior to participation” to minimize blue light interference on sleep assessments (38). After completion, responses were reviewed for missing data and implausible answers. When inconsistencies were identified, supplementary or repeated entries were requested to ensure completeness and accuracy. Data entry and cleaning were performed independently by two researchers and cross-validated by a third member before being compiled into a unified dataset.

**Figure 1:**
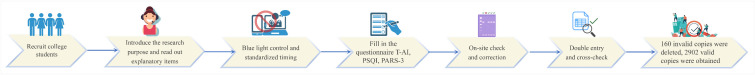
Test flow chart.

A total of 3,062 questionnaires were distributed. After excluding 160 invalid cases—25 with patterned responses, 42 completed in under 3 minutes, and 93 with values exceeding ±3 standard deviations — 2,902 valid questionnaires were retained (validity rate: 94.77%). Ethical approval for the study was obtained from the Ethics Committee of Shanghai University of Sport (Approval No. 102772021RT004). The procedural flow is illustrated in **Figure 1**.

### 2.3 Research instruments

#### 2.3.1 General information questionnaire

The General Information Questionnaire collects basic demographic data from participants, including age, gender, height, weight, family background, physical activity experience, and lifestyle habits.

#### 2.3.2 State-Trait Anxiety Inventory

The State-Trait Anxiety Inventory was developed and revised by Spielberger (39) and consists of two subscales: the State Anxiety Inventory (S-AI) and the Trait Anxiety Inventory (T-AI). Only the T-AI was used in this study. It comprises 20 items, including 11 positively scored and 9 negatively scored items. A four-point Likert scale was used: “1” indicates “hardly ever,” “2” indicates “somewhat,” “3” indicates “often,” and “4” indicates “almost always.” The total score was used as a predictor variable, with higher scores indicating greater levels of trait anxiety.

All psychological instruments used in this study followed the psychometric guidelines proposed by Guelmami et al. (2023) (40), ensuring that the tools demonstrated good reliability and validity.

#### 2.3.3 Pittsburgh Sleep Quality Index

The scale, developed by Buysse et al. (41) in 1989, contains 19 self-assessment items and 5 observer-assessment items. The 18 self-assessment items used in scoring encompass seven dimensions: sleep quality, time to fall asleep, sleep duration, sleep efficiency, sleep disturbance, use of sleeping medication, and daytime dysfunction. Each dimension is scored on a scale from 0 to 3, with a total score ranging from 0 to 21. The scores for each dimension were treated as outcome variables in this study, with higher scores indicating poorer sleep quality.

#### 2.3.4 Physical Activity Rating Scale

The scale was developed by Gongxiong Hashimoto and revised by Deqing Liang et al. (42). It comprises three indicators: exercise intensity, duration of each exercise session, and frequency of exercise. A five-point Likert scale is used to assess the level of physical activity among the participants, with higher total scores reflecting greater levels of physical activity.

The selection of measurement tools in this study aligns with established frameworks that call for methodological rigor and transparency in behavioral health research (43). Additionally, Dhahbi et al. (44, 45) provide valuable insights into the quantification of external training loads, which directly relate to the measurement tools used in our study and the interpretation of performance metrics.

To ensure appropriateness for the study population, face validity was assessed prior to formal data collection. A separate group of 20 college students who did not participate in the main study were invited to evaluate the clarity, wording, and perceived relevance of each item in the questionnaires. Based on their feedback, minor revisions were made to improve comprehension and suitability. This procedure is consistent with recommendations for improving scale usability in applied psychological assessments (46).

### 2.4 Data processing

Measurement data were presented as mean ± standard deviation, with results rounded to three decimal places. Before conducting group comparisons, the Shapiro–Wilk test and Levene’s test were used to examine normality and homogeneity of variance, respectively. The results confirmed that the variables met the assumptions required for ANOVA. One-way ANOVA was applied to compare sleep quality dimensions among college students with low, medium, and high levels of trait anxiety. Given the balanced group sizes, LSD *post hoc* tests were used to enhance sensitivity for pairwise comparisons. Categorical data were expressed as n (%) and analyzed using chi-square tests. Spearman correlation was used to assess the associations between trait anxiety levels and subcomponents of sleep quality.

To examine moderation effects, Model 1 of the PROCESS 4.0 macro was employed for multiple regression analysis. This model tested whether physical activity level, intensity, frequency, and duration moderated the relationship between sleep quality and trait anxiety. All variables were standardized prior to modeling. The regression output included unstandardized coefficients (β), standard errors (SE), t-values, p-values, and 95% confidence intervals (LLCI and ULCI).

All statistical inferences were two-tailed, with a significance threshold set at α = 0.05. Statistical significance was indicated as follows: * p < 0.05, ** p < 0.01, *** p < 0.001. All analyses were conducted using SPSS Statistics 26.0. To ensure the adequacy of statistical power, a *post hoc* power analysis was conducted. Assuming a medium effect size (Cohen’s f = 0.25), significance level α = 0.05, and three-group comparisons, the sample size of 2,902 yielded a statistical power of 1.0, indicating excellent sensitivity for detecting between-group differences.

## 3 Results

### 3.1 Differences in demographic variables across trait anxiety groups

Based on previous studies (47), trait anxiety was categorized into three groups: high trait anxiety was defined as one standard deviation above the mean score (21.754 + 4.121 ≈ 26), and low trait anxiety as one standard deviation below the mean (21.754 − 4.121 ≈ 18). The mean trait anxiety scores for the high, medium, and low groups were 55.72 ± 4.751, 42.47 ± 5.389, and 27.79 ± 3.336, respectively.

As shown in **Table 1**, a total of 2,902 college students participated in the study. Their mean BMI was 21.754 ± 4.121 kg/m², and the average age was 19.41 ± 1.555 years. Among them, 46.14% were male, 46.62% were only children, 9.06% were from single-parent families, 59.44% had urban household registration, 1.21% reported sports experience, 21.09% had a habit of drinking, 3.10% had a habit of smoking, and 83.60% engaged in social activities. Statistically significant differences were found across trait anxiety groups in age, gender, only-child status, single-parent family background, household registration type, and social activity participation (*p* < 0.05). No significant differences were observed in other demographic variables (all *p* > 0.05).

**Table 1:** Demographic differences across trait anxiety groups (n = 2,902).

Variables	Total (2, 902)	Groups of Trait Anxiety			Comparison between Groups	
		Low (*n* =504)	Medium (*n* =1996)	High (*n* =402)	*F (χ2)*	*P*
BMI (kg/m2)	21.754 ± 4.121	21.687 ± 4.193	21.834 ± 4.097	21.441 ± 4.143	1.602	0.202
Age (years)	19.41 ± 1.555	19.20 ± 1.283	19.45 ± 1.627	19.44 ± 1.487	5.319	0.005
Gender (male%)	46.14	47.42	47.29	38.80	10.104	0.006
Only Child (yes%)	46.62	53.37	45.94	41.54	13.769	0.001
Single-Parent Family (yes%)	9.06	8.93	8.17	13.68	12.363	0.002
Urban Household (yes%)	59.44	68.65	58.27	53.73	24.310	< 0.001
Sports Experience (yes%)	1.21	0.40	1.45	1.00	3.941	0.139
Drinking Habit (yes%)	21.09	18.65	21.09	24.13	4.033	0.133
Smoking Habit (yes%)	3.10	3.97	2.71	3.98	3.335	0.189
Social Activity (yes%)	83.60	90.87	84.37	70.65	69.493	< 0.001

BMI, Body Mass Index; F-values represent results of chi-square (χ²) tests as appropriate.

### 3.2 Differences in sleep quality among college students with varying levels of trait anxiety

One-way ANOVA revealed that all seven components of sleep quality—subjective sleep quality, sleep latency, sleep duration, sleep efficiency, sleep disturbances, use of sleeping medication, and daytime dysfunction—were significantly better in the low trait anxiety group compared to the medium and high trait anxiety groups (*p* < 0.05). Except for sleeping medication, which showed no statistically significant difference (*p* = 0.373), the remaining six components were significantly better in the medium trait anxiety group than in the high trait anxiety group (all *p* ≤ 0.01), as shown in **Table 2**.

**Table 2:** Differences in sleep quality by trait anxiety level.

Variables	Total (2, 902)	Groups of Trait Anxiety			Comparison between Groups		LSD *Post-hoc* Multiple Comparisons		
		Low (*n* =504)	Medium (*n* =1996)	High (*n* =402)	*F (χ2)*	*P*	Low VS Medium	Low VS High	Medium VS High
Sleep Quality	0.90 ± 0.721	0.55 ± 0.596	0.90 ± 0.688	1.34 ± 0.787	146.646	< 0.001	< 0.001	< 0.001	< 0.001
Time to Fall Asleep	1.50 ± 1.637	0.85 ± 1.260	1.51 ± 1.602	2.26 ± 1.876	87.731	< 0.001	< 0.001	< 0.001	< 0.001
Sleep Duration	0.66 ± 0.583	0.56 ± 0.553	0.66 ± 0.580	0.82 ± 0.600	21.624	< 0.001	0.001	< 0.001	< 0.001
Sleep Efficiency	0.37 ± 0.738	0.28 ± 0.662	0.36 ± 0.726	0.50 ± 0.863	9.341	< 0.001	0.031	< 0.001	0.001
Sleep Disturbance	0.81 ± 0.568	0.56 ± 0.531	0.81 ± 0.547	1.11 ± 0.570	110.883	< 0.001	< 0.001	< 0.001	< 0.001
Sleeping Medication	0.10 ± 0.404	0.05 ± 0.281	0.10 ± 0.410	0.12 ± 0.494	4.753	0.009	0.006	0.006	0.373
Daytime Dysfunction	1.12 ± 1.006	0.49 ± 0.713	1.10 ± 0.941	2.04 ± 0.971	324.863	< 0.001	< 0.001	< 0.001	< 0.001

F-values represent results of chi-square (χ²) tests as appropriate.

### 3.3 Correlation between trait anxiety and sleep dimensions

**Figure 2:**
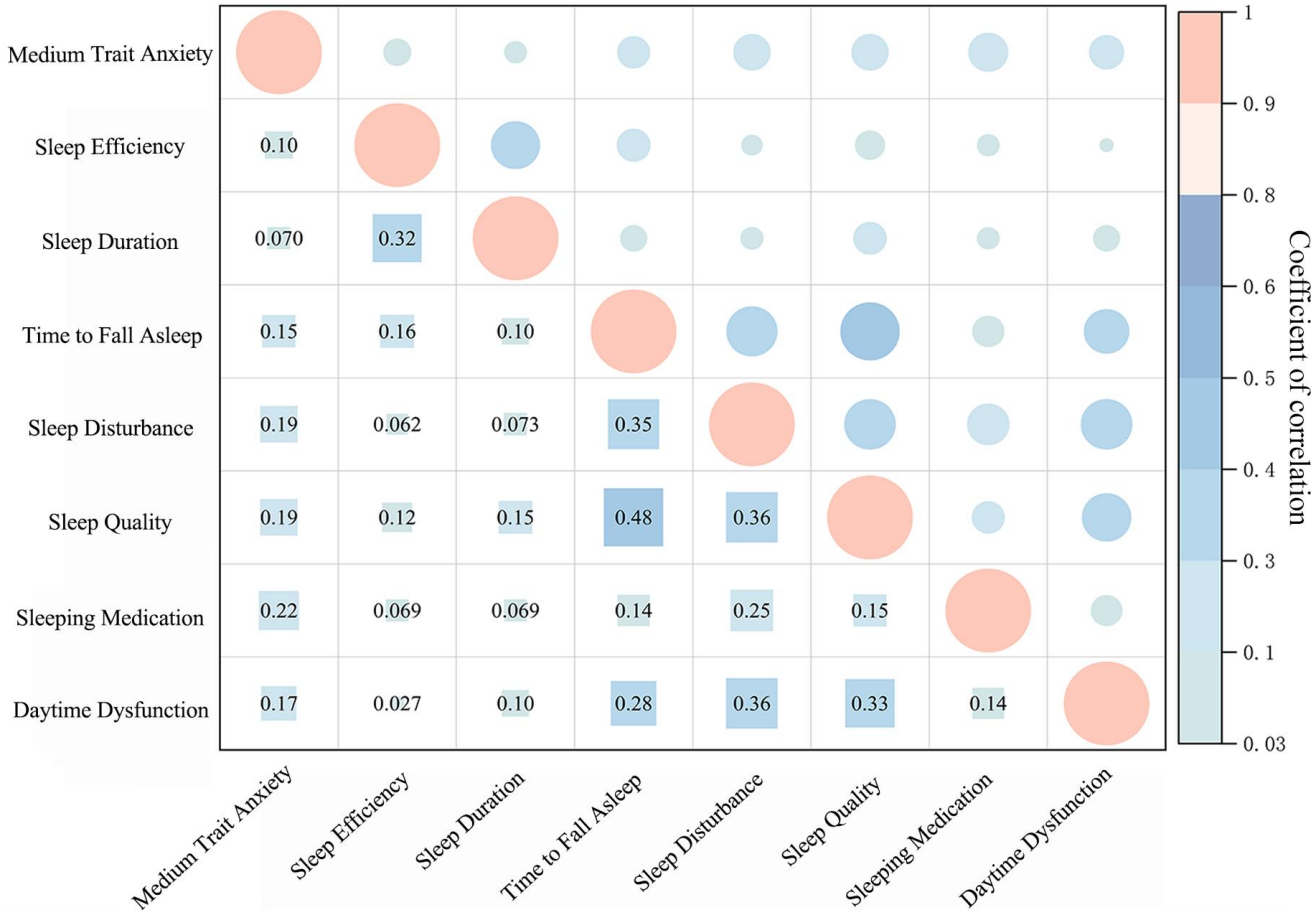
Heatmap of Spearman correlations between medium trait anxiety and sleep subdimensions among college students. Color gradients from red to blue to green (from dark to light) represent decreasing correlation strength. The larger the shape, the stronger the correlation. All coefficients shown are significant at *p* < 0.05.

Spearman correlation coefficient were used to examine the relationship between low, medium, and high levels of trait anxiety and various dimensions of sleep quality. As shown in **Table 3** and **Figure 2**, low trait anxiety was significantly positively correlated with scores for time to fall asleep (r = 0.149, *p* < 0.001), sleep disturbance (r = 0.137, *p* < 0.001), sleep quality (r = 0.188, *p* < 0.001), and daytime dysfunction (r = 0.106, *p* < 0.05). Medium trait anxiety was significantly positively correlated with sleep efficiency (r = 0.102, *p* < 0.001), sleep duration (r = 0.070, *p* < 0.001), time to fall asleep (r = 0.154, *p* < 0.001), sleep disturbance (r = 0.187, *p* < 0.001), sleep quality (r = 0.192, *p* < 0.001), sleeping medication (r = 0.216, *p* < 0.001), and daytime dysfunction (r = 0.170, *p* < 0.001). High trait anxiety also showed significant positive associations with sleep duration (r = 0.170, *p* < 0.001), time to fall asleep (r = 0.200, *p* < 0.001), sleep disturbance (r = 0.249, *p* < 0.001), sleep quality (r = 0.170, *p* < 0.001), and daytime dysfunction (r = 0.264, *p* < 0.001). These findings suggest that students with high levels of trait anxiety experienced more pronounced impairments in sleep components than those with low trait anxiety, though less severe than those with medium trait anxiety. The medium trait anxiety group exhibited the highest prevalence of sleep disturbances among the three groups.

**Table 3:** Correlation between trait anxiety levels and sleep quality components.

Variables	Low Trait Anxiety	Medium Trait Anxiety	High Trait Anxiety
Sleep Efficiency	0.076	0.102**	0.067
Sleep Duration	0.011	0.070**	0.170**
Time to Fall Asleep	0.149**	0.154**	0.200**
Sleep Disturbance	0.137**	0.187**	0.249**
Sleep Quality	0.188**	0.192**	0.170**
Sleeping Medication	0.057	0.216**	-0.047
Daytime Dysfunction	0.106*	0.170**	0.264**

*p* < 0.05 (*), *p* < 0.01 (**).

### 3.4 Moderating effect of physical activity level on the relationship between trait anxiety and sleep quality

A significant positive correlation was observed between trait anxiety and sleep factor scores. To further examine whether varying levels of physical activity (PA) moderated this relationship, moderation regression models were constructed (**Table 4**), following the steps for testing moderating effects outlined by Wen Zhonglin (48). The seven sleep factor scores (sleep quality, time to fall asleep, sleep duration, sleep efficiency, sleep disturbance, sleeping medication, and daytime dysfunction) were used as predictor variables, trait anxiety as the outcome variable, and total PA level as the moderator.

**Table 4:** Regression models of different physical activity indicators moderating the relationship between trait anxiety and sleep factors.

Model	*β*	se	*t*	*p*	LLCI	ULCI
constant	42.805	2.054	20.839	< 0.001	38.777	46.832
Sleep Quality	4.177	0.222	18.821	< 0.001	3.742	4.612
Different Levels of PA	-0.048	0.01	-4.914	< 0.001	-0.067	-0.029
Different Levels of PA*Sleep Quality	0.03	0.012	2.558	0.011	0.007	0.053
constant	41.95	2.101	19.97	< 0.001	37.831	46.069
Time to Fall Asleep	1.459	0.1	14.571	< 0.001	1.262	
Different Levels of PA	-0.054	0.01	-5.394	< 0.001	-0.073	-0.034
Different Levels of PA*Time to Fall Asleep	0.012	0.006	2.12	0.034	0.001	0.023
constant	39.596	2.158	18.349	< 0.001	35.364	43.827
Sleep Efficiency	1.327	0.228	5.809	< 0.001	0.879	1.775
Different Levels of PA	-0.052	0.01	-5.055	< 0.001	-0.072	-0.032
Different Levels of PA*Sleep Efficiency	0.036	0.013	2.765	0.006	0.011	0.062
constant	41.206	1.968	20.937	< 0.001	37.347	45.065
Daytime Dysfunction	3.852	0.153	25.146	< 0.001	3.552	4.153
Different Levels of PA	-0.044	0.009	-4.664	< 0.001	-0.062	-0.025
Different Levels of PA*Daytime Dysfunction	0.02	0.009	2.208	0.027	0.002	0.038
constant	42.817	2.056	20.821	< 0.001	38.785	46.849
Sleep Quality	4.212	0.222	18.957	< 0.001	3.776	4.648
Different PA Intensities	-1.017	0.241	-4.223	< 0.001	-1.489	-0.545
Different PA Intensities*Sleep Quality	0.677	0.318	2.125	0.034	0.052	1.301
constant	39.789	2.161	18.414	< 0.001	35.552	44.026
Sleep Efficiency	1.326	0.228	5.816	< 0.001	0.879	1.773
Different PA Intensities	-1.125	0.254	-4.43	< 0.001	-1.623	-0.627
Different PA Intensities*Sleep Efficiency	0.727	0.329	2.208	0.027	0.081	1.372
constant	41.293	1.968	20.978	< 0.001	37.434	45.153
Daytime Dysfunction	3.875	0.153	25.294	< 0.001	3.574	4.175
Different PA Intensities	-1.058	0.231	-4.58	< 0.001	-1.511	-0.605
Different PA Intensities*Daytime Dysfunction	0.594	0.225	2.635	0.008	0.152	1.035
constant	39.91	2.161	18.465	< 0.001	35.672	44.148
Sleep Efficiency	1.363	0.228	5.981	< 0.001	0.916	1.81
Different PA Durations	-0.784	0.227	-3.455	0.001	-1.229	-0.339
Different PA Durations*Sleep Efficiency	0.879	0.313	2.808	0.005	0.265	1.492
constant	43.476	2.052	21.185	< 0.001	39.452	47.5
Sleep Quality	4.251	0.222	19.169	< 0.001	3.816	4.685
Different PA Frequencies	-1.23	0.288	-4.271	< 0.001	-1.795	-0.666
Different PA Frequencies*Sleep Quality	1.309	0.386	3.395	0.001	0.553	2.066
constant	42.556	2.103	20.238	< 0.001	38.433	46.679
Time to Fall Asleep	1.468	0.1	14.635	< 0.001	1.271	1.664
Different PA Frequencies	-1.151	0.295	-3.899	< 0.001	-1.729	-0.572
Different PA Frequencies*Time to Fall Asleep	0.508	0.176	2.882	0.004	0.162	0.854
constant	41.769	1.963	21.277	< 0.001	37.919	45.618
Daytime Dysfunction	3.902	0.153	25.545	< 0.001	3.602	4.201
Different PA Frequencies	-1.328	0.277	-4.802	< 0.001	-1.87	-0.786
Different PA Frequencies*Daytime Dysfunction	0.996	0.269	3.697	< 0.001	0.468	1.524

PA refers to Physical Activity. Only statistically significant variables (*p* < 0.05) in the moderation regression models are presented; non-significant predictors were omitted for simplicity.

**Figure 3:**
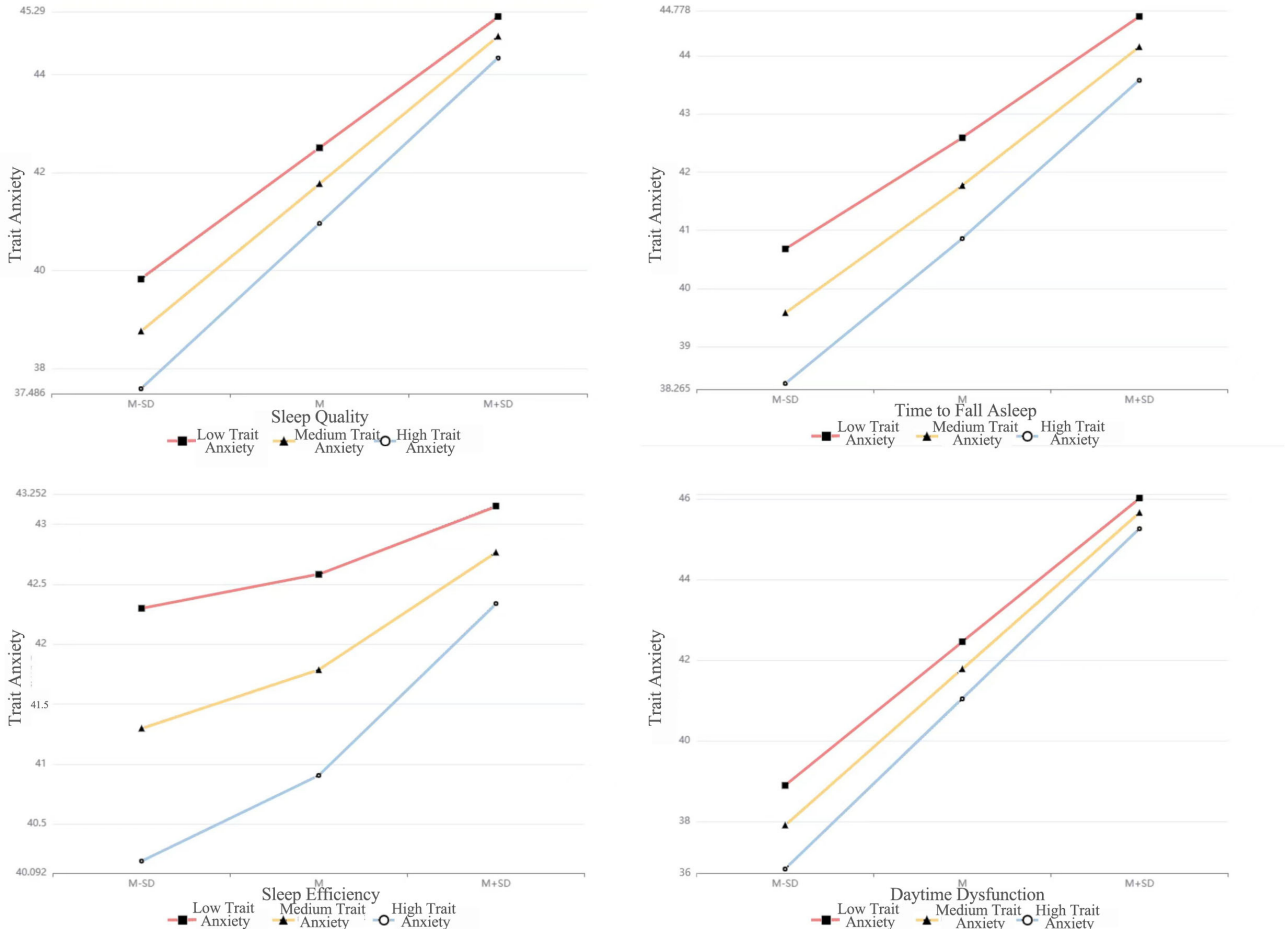
Moderating effects of different physical activity levels on the relationship between trait anxiety and sleep factors.

After including covariates with statistically significant univariate results, the explanatory power (*R²*) of the models for trait anxiety was 16.3%, 12.4%, 7.6%, 7.1%, 38.4%, 7.2%, and 22.7%, respectively—all statistically significant (*p* < 0.001). The moderating effects of low, medium, and high levels of PA showed a linearly increasing trend (**Figure 3**, **Table 5**).

**Table 5:** Moderating effects of physical activity level, intensity, duration, and frequency on the relationship between trait anxiety and sleep quality.

Model	*β*	se	*t*	*p*	LLCI	ULCI
constant	43.476	2.052	21.185	< 0.001	39.452	47.500
Sleep Quality	4.251	0.222	19.169	< 0.001	3.816	4.685
Different PA Frequencies	-1.230	0.288	-4.271	< 0.001	-1.795	-0.666
Different PA Frequencies * Sleep Quality	1.309	0.386	3.395	0.001	0.553	2.066
Gender	-0.590	0.321	-1.837	0.066	-1.220	0.040
Age	0.126	0.103	1.229	0.219	-0.075	0.327
Only Child (yes%)	-0.675	0.366	-1.846	0.065	-1.392	0.042
Single-Parent Family (yes%)	0.880	0.557	1.581	0.114	-0.211	1.971
Urban Household (yes%)	-1.620	0.370	-4.378	< 0.001	-2.345	-0.894
Social Activity (yes%)	-3.233	0.432	-7.485	< 0.001	-4.080	-2.386
constant	42.556	2.103	20.238	< 0.001	38.433	46.679
Time to Fall Asleep	1.468	0.100	14.635	< 0.001	1.271	1.664
Different PA Frequencies	-1.151	0.295	-3.899	< 0.001	-1.729	-0.572
Different PA Frequencies * Time to Fall Asleep	0.508	0.176	2.882	0.004	0.162	0.854
Gender	-0.332	0.330	-1.008	0.313	-0.979	0.314
Age	0.176	0.105	1.669	0.095	-0.031	0.382
Only Child (yes%)	-0.660	0.375	-1.762	0.078	-1.395	0.074
Single-Parent Family (yes%)	1.088	0.570	1.908	0.056	-0.030	2.206
Urban Household (yes%)	-1.794	0.379	-4.733	< 0.001	-2.538	-1.051
Social Activity (yes%)	-3.319	0.443	-7.489	< 0.001	-4.188	-2.450
constant	40.405	2.163	18.678	< 0.001	36.163	44.647
Sleep Efficiency	1.334	0.231	5.766	< 0.001	0.880	1.788
Different PA Frequencies	-1.008	0.306	-3.294	0.001	-1.607	-0.408
Different PA Frequencies * Sleep Efficiency	0.577	0.375	1.539	0.124	-0.158	1.313
Gender	-0.547	0.340	-1.609	0.108	-1.214	0.120
Age	0.322	0.108	2.986	0.003	0.111	0.534
Only Child (yes%)	-0.830	0.387	-2.147	0.032	-1.589	-0.072
Single-Parent Family (yes%)	1.005	0.589	1.706	0.088	-0.150	2.160
Urban Household (yes%)	-1.836	0.392	-4.688	< 0.001	-2.604	-1.068
Social Activity (yes%)	-3.885	0.455	-8.535	< 0.001	-4.777	-2.992
constant	41.769	1.963	21.277	< 0.001	37.919	45.618
Daytime Dysfunction	3.902	0.153	25.545	< 0.001	3.602	4.201
Different PA Frequencies	-1.328	0.277	-4.802	< 0.001	-1.870	-0.786
Different PA Frequencies * Daytime Dysfunction	0.996	0.269	3.697	< 0.001	0.468	1.524
Gender	0.189	0.310	0.609	0.543	-0.419	0.796
Age	0.174	0.098	1.778	0.076	-0.018	0.367
Only Child (yes%)	-0.614	0.351	-1.750	0.080	-1.302	0.074
Single-Parent Family (yes%)	0.984	0.534	1.843	0.066	-0.063	2.031
Urban Household (yes%)	-1.778	0.355	-5.009	< 0.001	-2.474	-1.082
Social Activity (yes%)	-2.677	0.415	-6.443	< 0.001	-3.492	-1.862

PA refers to Physical Activity. Only statistically significant variables (p < 0.05) in the moderation regression models are presented; non-significant predictors were omitted for simplicity.

All seven sleep factors were significant positive predictors of trait anxiety (*p* < 0.001), while PA levels were significant negative predictors (*p* < 0.001). The interaction terms between PA level and four specific sleep factors—sleep quality, time to fall asleep, sleep efficiency, and daytime dysfunction—were significant (*p* < 0.05), indicating that PA positively moderated the relationship between these sleep factors and trait anxiety. The changes in *R²* for these moderation effects were 0.002, 0.001, 0.002, and 0.001, respectively, suggesting that the moderation contributed 0.2%, 0.1%, 0.2%, and 0.1% of the variance. As illustrated in **Figure 3**, higher PA levels were associated with stronger moderation effects, potentially enhancing the link between sleep and anxiety.

Moreover, urban household registration and engagement in social activities significantly negatively moderated the relationship between PA and trait anxiety across multiple sleep factors (*p* < 0.001). Single-parent family status significantly positively influenced the effect of PA on the relationship between trait anxiety and time to fall asleep (*p* = 0.048). Age significantly positively moderated the relationship between PA and both sleep efficiency (*p* = 0.001) and daytime dysfunction (*p* = 0.047) (**Table 6**).

**Table 6:** Covariate effects of demographic variables in moderation models.

Moderator Variables	Sleep Factors	Model	*β*	se	*t*	*p*	LLCI	ULCI
Different Levels of PA	Sleep Quality	Urban Household (yes%)	-1.552	0.37	-4.191	< 0.001	-2.278	-0.826
	Sleep Quality	Social Activity (yes%)	-3.104	0.434	-7.144	< 0.001	-3.955	-2.252
	Time to Fall Asleep	Single-Parent Family (yes%)	1.126	0.569	1.979	0.048	0.01	2.242
	Time to Fall Asleep	Urban Household (yes%)	-1.709	0.379	-4.511	< 0.001	-2.452	-0.966
	Time to Fall Asleep	Social Activity (yes%)	-3.142	0.445	-7.054	< 0.001	-4.015	-2.268
	Sleep Efficiency	Age	0.342	0.108	3.185	0.001	0.132	0.553
	Sleep Efficiency	Urban Household (yes%)	-1.768	0.391	-4.525	< 0.001	-2.534	-1.002
	Sleep Efficiency	Social Activity (yes%)	-3.671	0.456	-8.047	< 0.001	-4.565	-2.77
	Daytime Dysfunction	Age	0.195	0.098	1.988	0.047	0.003	0.388
	Daytime Dysfunction	Urban Household (yes%)	-1.705	0.356	-4.793	< 0.001	-2.403	-1.008
	Daytime Dysfunction	Social Activity (yes%)	-2.635	0.418	-6.296	< 0.001	-3.455	-1.814
Different PA Intensities	Sleep Quality	Urban Household (yes%)	-1.562	0.371	-4.212	< 0.001	-2.289	-0.835
	Sleep Quality	Social Activity (yes%)	-3.129	0.434	-7.206	< 0.001	-3.981	-2.278
	Sleep Efficiency	Age	0.34	0.108	3.161	0.002	0.129	0.552
	Sleep Efficiency	Only Child (yes%)	-0.781	0.386	-2.021	0.043	-1.538	-0.023
	Sleep Efficiency	Urban Household (yes%)	-1.797	0.391	-4.591	< 0.001	-2.565	-1.03
	Sleep Efficiency	Social Activity (yes%)	-3.734	0.456	-8.18	< 0.001	-4.629	-2.839
	Daytime Dysfunction	Age	0.194	0.098	1.978	0.048	0.002	0.387
	Daytime Dysfunction	Urban Household (yes%)	-1.705	0.356	-4.79	< 0.001	-2.403	-1.007
	Daytime Dysfunction	Social Activity (yes%)	-2.655	0.418	-6.352	< 0.001	-3.475	-1.835
Different PA Durations	Sleep Efficiency	Age	0.339	0.108	3.145	0.002	0.128	0.55
	Sleep Efficiency	Only Child (yes%)	-0.822	0.386	-2.129	0.033	-1.579	-0.065
	Sleep Efficiency	Urban Household (yes%)	-1.764	0.392	-4.507	< 0.001	-2.532	-0.997
	Sleep Efficiency	Social Activity (yes%)	-3.819	0.456	-8.383	< 0.001	-4.712	-2.926
Different PA Frequencies	Sleep Quality	Urban Household (yes%)	-1.62	0.37	-4.378	< 0.001	-2.345	-0.894
	Sleep Quality	Social Activity (yes%)	-3.233	0.432	-7.485	< 0.001	-4.08	-2.386
	Time to Fall Asleep	Urban Household (yes%)	-1.794	0.379	-4.733	< 0.001	-2.538	-1.051
	Time to Fall Asleep	Social Activity (yes%)	-3.319	0.443	-7.489	< 0.001	-4.188	-2.45
	Daytime Dysfunction	Urban Household (yes%)	-1.778	0.355	-5.009	< 0.001	-2.474	-1.082
	Daytime Dysfunction	Social Activity (yes%)	-2.677	0.415	-6.443	< 0.001	-3.492	-1.862

PA refers to Physical Activity. Only statistically significant variables (p < 0.05) in the moderation regression models are presented; non-significant predictors were omitted for simplicity.

### 3.5 Moderating effect of physical activity intensity

**Figure 4:**
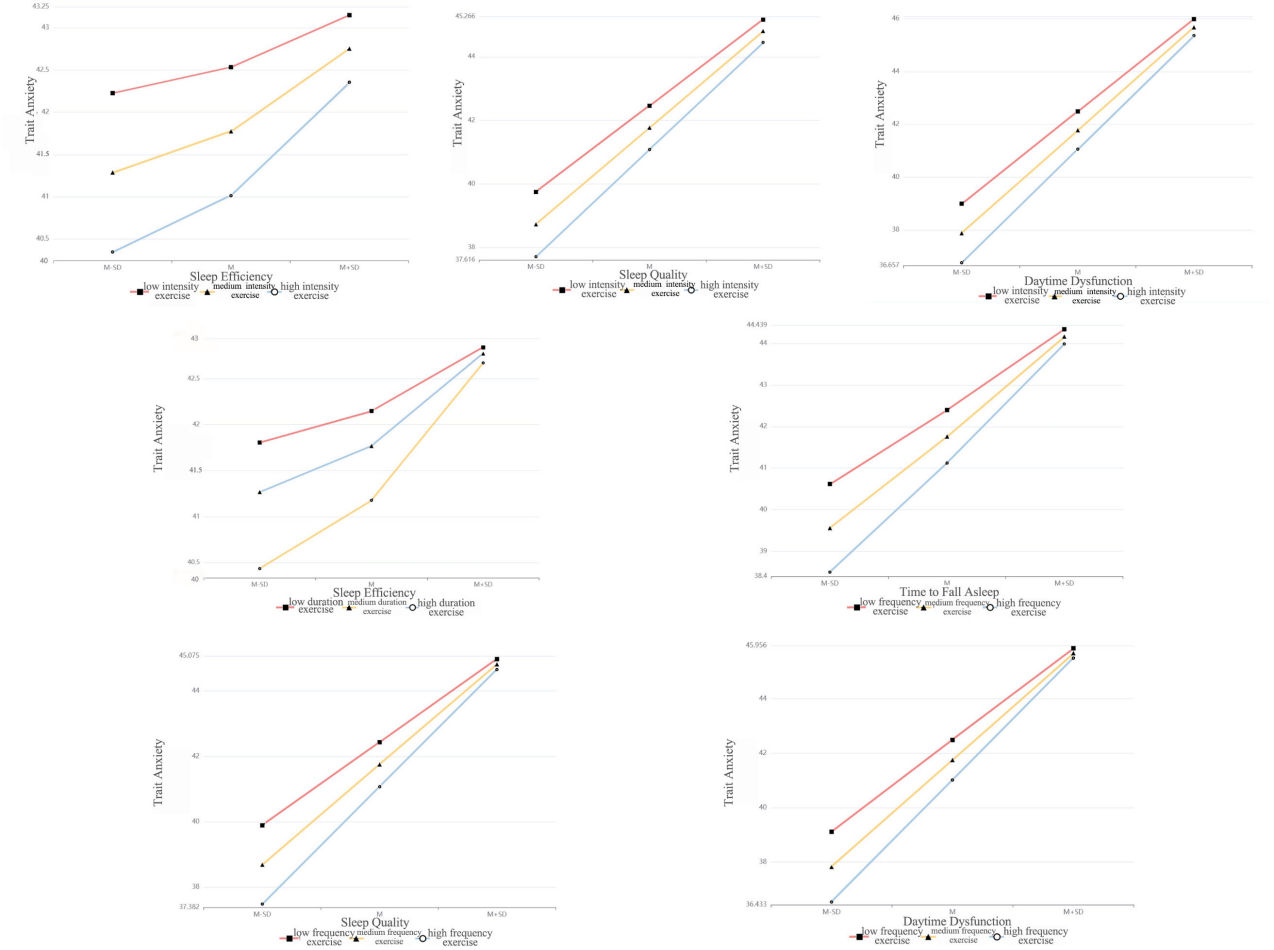
Moderating effects of different physical activity components on the relationship between trait anxiety and sleep quality.

To further examine whether different intensities, durations, and frequencies of physical activity could moderate the relationship between trait anxiety and sleep factor scores, regression models were constructed by including trait anxiety as the predictor, sleep factor scores as the outcomes, and incorporating physical activity metrics and statistically significant covariates. The results showed that the explanatory power (*R²*) of sleep quality, time to fall asleep, sleep efficiency, and daytime dysfunction scores by different physical activity intensities on trait anxiety were 16.1%, 12.0%, 6.7%, and 22.7%, respectively, all statistically significant (*p* < 0.001). The moderating effect of low, medium, and high physical activity intensities showed a linearly increasing trend (**Figure 4**, **Table 5**).

All four sleep factor scores were significant positive predictors of trait anxiety (*p* < 0.001), whereas physical activity intensity was a significant negative predictor (*p* < 0.05). Furthermore, the interaction terms of sleep quality, sleep efficiency, and daytime dysfunction scores with physical activity intensity were also significant positive predictors of trait anxiety (*p* < 0.05). However, the interaction between time to fall asleep and physical activity intensity was not statistically significant (*p* = 0.526). These findings indicate that physical activity intensity positively moderates the relationship between trait anxiety and sleep quality, sleep efficiency, and daytime dysfunction scores. The changes in *R²* for these interactions were 0.001, 0.002, and 0.002, respectively, suggesting that the moderating effects contributed 0.1%, 0.2%, and 0.2% of the variance. As illustrated in **Figure 4**, physical activity intensity positively moderated the relationship between trait anxiety and sleep quality, sleep efficiency, and daytime dysfunction scores, potentially enhancing their associations.

In addition, the predictive effects of physical activity intensity on the relationship between trait anxiety and the scores of sleep quality, sleep efficiency, and daytime dysfunction were significantly negatively influenced by both urban residence and engagement in social activities (all *p* < 0.001). Age was a significant positive moderator in the relationship between physical activity intensity and the scores of sleep efficiency (*p* = 0.002) and daytime dysfunction (*p* = 0.048). Moreover, being an only child significantly negatively moderated the association between physical activity intensity and sleep efficiency (*p* = 0.043) (**Table 6**).

### 3.6 Moderating effect of physical activity duration

The results indicated that the explanatory degrees (*R²*) of sleep quality, time to fall asleep, sleep efficiency, and daytime dysfunction scores by different physical activity durations on trait anxiety were 15.8%, 11.9%, 6.6%, and 22.3%, respectively, all statistically significant (*p* < 0.001). The moderating effect of low, medium, and high physical activity durations exhibited a linearly increasing trend (**Figure 4**, **Table 5**).

All four sleep factor scores were significant positive predictors of trait anxiety (*p* < 0.001), while different physical activity durations were significant negative predictors (*p* < 0.001). Furthermore, the interaction between sleep efficiency and physical activity duration was a significant positive predictor of trait anxiety (*p* = 0.005). However, the interactions involving sleep quality, time to fall asleep, and daytime dysfunction were not statistically significant (*p* > 0.05). These findings suggest that physical activity duration positively moderated the relationship between sleep efficiency and trait anxiety. The change in *R²* was 0.003, indicating that the moderation effect accounted for 0.3% of the variance. As illustrated in **Figure 4**, physical activity duration exhibited a positive moderating effect on the relationship between trait anxiety and sleep efficiency, potentially enhancing their association.

In addition, the moderating effect of physical activity duration on the relationship between trait anxiety and sleep efficiency was significantly negatively influenced by urban residence (*p* < 0.001), social activity engagement (*p* < 0.001), and being an only child (*p* = 0.033), while age showed a significant positive influence (*p* = 0.002) (**Table 6**).

### 3.7 Moderating effect of physical activity frequency

The results indicated that the explanatory degrees (*R²*) of sleep quality, time to fall asleep, sleep efficiency, and daytime dysfunction scores by different physical activity frequencies on trait anxiety were 16.3%, 12.1%, 6.4%, and 22.9%, respectively, all statistically significant (*p* < 0.001). The moderating effects of low-, medium-, and high-frequency physical activity exhibited a linearly increasing trend (**Figure 4**, **Table 5**).

All four sleep factors were significant positive predictors of trait anxiety (*p* < 0.001), while physical activity frequency was a significant negative predictor (*p* ≤ 0.001). Moreover, the interaction terms between physical activity frequency and sleep quality, time to fall asleep, and daytime dysfunction scores were significant positive predictors of trait anxiety (*p* < 0.05). However, the interaction term between sleep efficiency and physical activity frequency was not statistically significant (*p* = 0.124). These results suggest that physical activity frequency positively moderated the relationships between trait anxiety and sleep quality, time to fall asleep, and daytime dysfunction. The changes in *R²* were 0.003, 0.003, and 0.004, indicating that the moderation effects contributed to 0.3%, 0.3%, and 0.4% of the variance, respectively. As shown in **Figure 4**, higher physical activity frequency enhanced the associations between trait anxiety and the corresponding sleep factors.

Furthermore, the moderating effect of physical activity frequency on the relationship between trait anxiety and sleep quality, time to fall asleep, and daytime dysfunction was significantly negatively influenced by urban residence and social activity engagement (all *p* < 0.001).

## 4 Discussion

This study found that physical activity exerted a significant dose-dependent moderating effect on the relationship between trait anxiety and sleep quality, which aligns with previous research findings (1, 2). The results showed that exercise intensity, duration, and frequency moderated the relationship between trait anxiety and different sleep subdimensions. Specifically, different levels of physical activity positively moderated the associations between sleep quality, time to fall asleep, sleep efficiency, daytime dysfunction scores, and trait anxiety, potentially enhancing their interaction. High-intensity physical activity had the most pronounced moderating effect on the associations between trait anxiety and sleep efficiency, sleep quality, and daytime dysfunction. Longer exercise durations were associated with stronger moderating effects on trait anxiety and sleep efficiency, particularly when the duration exceeded 60 minutes. More frequent physical activity showed stronger moderating effects on trait anxiety and sleep quality, sleep latency, and daytime dysfunction, with the most significant effects observed in those exercising more than three times per week. These results are consistent with the findings of Korman et al. (49), who suggested that high-intensity interval training benefits both cardiorespiratory health and emotional regulation. Their experimental study also supported the value of high-frequency exercise (25), demonstrating that adolescents who engaged in running every weekday for three consecutive weeks experienced significant improvements in both subjective and objective sleep metrics, along with enhanced psychological functioning. The current findings extend this evidence to the college student population, highlighting that high-intensity, long-duration, and high-frequency physical activity not only improves sleep but also plays a crucial role in anxiety regulation. This dose-dependent effect may be driven by several neurobiological mechanisms. On one hand, physical activity can regulate hypothalamic–pituitary–adrenal (HPA) axis function, reducing abnormal cortisol secretion caused by trait anxiety, thus alleviating hyperarousal and sympathetic overactivation (50). On the other hand, exercise can increase the release of serotonin (5-HT) and endorphins, thereby improving emotional state and sleep quality. Notably, the rise and subsequent rapid drop in body temperature after high-intensity exercise may activate deep relaxation mechanisms, facilitating the onset of deep sleep (51). Therefore, adequately dosed physical activity may serve as a promising strategy for anxiety intervention and sleep promotion.

Social psychology research suggests that completing high-intensity physical activity can enhance individual psychological resilience and self-efficacy, facilitating the positive transformation of negative mental states (52). High-intensity exercise helps college students with trait anxiety better alleviate anxiety, improve sleep efficiency and quality, and reduce daytime dysfunction. In contrast, low-intensity exercise may be insufficient to trigger the physiological and psychological changes necessary for improving mood and sleep (49), indicating that sufficient exercise load is essential for reducing trait anxiety. However, exercise intensity should be adjusted based on individual physical conditions to avoid excessive fatigue and negative emotions caused by overexertion. These findings further support the World Health Organization’s physical activity guidelines (53), suggesting the need for future integration of evidence from both biological and behavioral research. Moreover, sustained physical activity can help anxious students shift attention away from unpleasant stimuli or physical discomfort, thereby improving their overall emotional state. Long-duration exercise can also regulate and extend slow-wave sleep (SWS), which is particularly important for memory consolidation, physical recovery, and emotional regulation (54). Continuous exercise not only enhances central nervous system function and regulates monoamine neurotransmitters and their receptors, but also improves physical fitness, blood circulation, and cardiopulmonary function, optimizing oxygen delivery during sleep and thereby improving sleep efficiency. Notably, some studies suggest that exercise durations of 20–60 minutes are most beneficial for emotional improvement (2). This difference may be due to the physical capabilities of college students, who may require longer activity durations to experience the same benefits. Additionally, the benefits of physical activity may involve delayed and threshold effects, suggesting that very short durations may not fully produce the intended positive outcomes. Converging evidence also shows that exercise can reduce negative emotions and maintain its effects for up to 24 hours after cessation (2). The regularization of physical activity behavior promotes gradual adaptation and cumulative benefits, supports the development of structured routines, and fosters a more positive lifestyle.

In addition to the dose–response effects, this study verified the significant positive correlations between trait anxiety and the subdimensions of sleep quality, particularly among college students with moderate to high levels of trait anxiety, who exhibited more pervasive sleep problems. Students with low trait anxiety consistently scored better across all sleep subdimensions than those in the moderate trait anxiety group, while the moderate group, except for the use of sleeping medication, outperformed the high trait anxiety group. These findings are supported by previous epidemiological studies (20, 55, 56), which have demonstrated that anxiety exerts a direct and substantial impact on sleep quality, independent of comorbid depression (57). Higher anxiety levels are often associated with excessive sympathetic nervous system activation, resulting in prolonged sleep latency, frequent nocturnal awakenings, reduced deep sleep, and increased daytime dysfunction, all of which impair sleep quality and contribute to sleep disorders (58). Furthermore, the manifestations of sleep disorders differ depending on the severity of anxiety. Sleep and anxiety share overlapping neural networks; with increasing anxiety, brain regions responsible for emotion and stress regulation—such as the amygdala, insula, and anterior cingulate cortex—exhibit hyperactivation, further exacerbating sleep disturbances (59, 60). Simultaneously, decreased connectivity between the medial prefrontal cortex (mPFC) and the amygdala reflects impaired top-down regulation, which not only worsens anxiety symptoms but also alters sleep architecture and degrades sleep quality (61). These problems often form a negative feedback loop, wherein individuals with high anxiety levels worry excessively about insufficient sleep and its consequences, resulting in greater emotional distress and maladaptive behaviors that interfere with sleep onset and maintenance (62). Prolonged psychological stress may also reduce the duration of slow-wave sleep (SWS) (63), thereby lowering sleep efficiency, shortening total sleep time, and gradually increasing reliance on sleeping medications. However, the low trait anxiety group reported fewer instances of sleeping medication use compared to the moderate and high anxiety groups, between whom there was no notable difference. This may be because individuals with lower anxiety experience milder sleep disturbances and are thus less dependent on pharmacological aids, while those in the moderate and high anxiety groups face comparable physiological and psychological stress, leading to similar levels of medication reliance. This finding highlights the importance of implementing comprehensive intervention strategies when addressing sleep problems in individuals with moderate or high trait anxiety, with the aim of reducing reliance on sleeping medications, improving sleep quality, alleviating anxiety symptoms, and minimizing potential drug-related risks.

Sleep quality is a critical factor associated with trait anxiety in college students, and its decline may serve as an independent predictor of psychiatric disorders. Neurobiological studies have shown that as trait anxiety increases, the level of autonomic nervous system activation also rises, leading to dysregulation of the endocrine system and promoting the release of stress hormones such as adrenaline and cortisol. This hormonal imbalance disrupts central nervous system function and alters sleep architecture. Anxiety is also known to disrupt neurotransmitters such as glutamate and gamma-aminobutyric acid (GABA), further weakening effective communication between the central and peripheral systems (13). Moreover, in individuals with trait anxiety, the levels of pro-inflammatory cytokines increase with rising anxiety, amplifying inflammatory responses and affecting the production of critical hormones such as serotonin and melatonin (64). These hormones play a vital role in maintaining regular sleep cycles and may impact sleep onset latency, sleep duration, sleep efficiency, nocturnal awakenings, and daytime functioning. As a result, the restorative functions of sleep are impaired. In turn, sleep disturbances damage neural plasticity and stress-related immune pathways, potentially leading to exacerbated anxiety symptoms or other psychiatric conditions (55, 65, 66). Therefore, to prevent the onset and progression of trait anxiety and sleep disorders, more attention should be directed toward monitoring sleep quality in individuals with elevated anxiety. Particular emphasis should be placed on tracking the use of hypnotic medications as an early warning sign.

The significance of this study lies in its exploration of physical activity as a moderating variable in the relationship between various dimensions of sleep quality and trait anxiety. The findings clarify how different levels, intensities, durations, and frequencies of physical activity influence the association between trait anxiety and specific dimensions of sleep quality. Additionally, the study reveals how sociodemographic factors shape the moderating effects of physical activity. These results underscore the importance of the “dose-response effect” of exercise in emotional research and contribute to refining the behavioral mechanisms by which exercise promotes mental health. However, as this study employed a cross-sectional design, the moderating effects of physical activity on sleep quality and trait anxiety cannot confirm causal relationships. It remains possible that pre-existing sleep problems or high levels of anxiety may inhibit an individual’s motivation and willingness to engage in physical activity, thereby influencing their activity levels (67). Future research is encouraged to adopt longitudinal tracking or experimental intervention designs to further elucidate the causal pathways and dynamic relationships among physical activity, sleep quality, and trait anxiety over time.

## 5 Shortcomings and prospects

1. The study employed a cross-sectional design, which precludes causal inference. The current findings cannot determine the directional relationship between physical activity, sleep quality, and trait anxiety. Further longitudinal studies are required to gain a deeper understanding of the time-varying relationships and associated effect sizes between physical activity, sleep quality, and trait anxiety.
2. The current study only examined sleep quality and the level, intensity, duration, and frequency of physical activity through a self-report questionnaire, which may have introduced some subjective bias. In particular, pre-existing sleep problems or high anxiety may reduce an individual’s willingness or motivation to engage in physical activity, thereby influencing the reported exercise level. It is recommended that future studies consider specific forms of exercise and use measurement tools such as heart rate monitors and accelerometers to more objectively assess physical activity, as well as EEG diagnostics and other methods to more accurately assess sleep quality and trait anxiety symptoms.
3. It is important to note that physical activity habits and mental health status of college students are inevitably affected by the social environment. Therefore, the generalizability of the findings may be limited. Further analysis and cross-population comparison are necessary to validate the applicability of these findings to different groups.

## 6 Conclusions and recommendations

Higher levels of trait anxiety are associated with poorer overall sleep quality and greater impairments across all sleep subdimensions among college students. Physical activity significantly alleviates anxiety and improves sleep, with stronger moderating effects observed at higher intensity, longer duration, and greater frequency. This study highlights the “dose-response effect” of physical activity and recommends that universities promote high-intensity, long-duration, and frequent exercise interventions to prevent and address mental health issues.

## Data Availability

The raw data supporting the conclusions of this article will be made available by the authors, without undue reservation.
